# Insights into *Solea senegalensis* Reproduction Through Gonadal Tissue Methylation Analysis and Transcriptomic Integration

**DOI:** 10.3390/biom15010054

**Published:** 2025-01-02

**Authors:** Daniel Ramírez, Marco Anaya-Romero, María Esther Rodríguez, Alberto Arias-Pérez, Robert Mukiibi, Helena D’Cotta, Diego Robledo, Laureana Rebordinos

**Affiliations:** 1 Área de Genética, Facultad de Ciencias del Mar y Ambientales, INMAR, Universidad de Cádiz, 11510 Cádiz, Spain; daniel.ramirez@uca.es (D.R.); alberto.arias@uca.es (A.A.-P.);; 2 The Roslin Institute and Royal (Dick) School of Veterinary Studies, University of Edinburgh, Edinburgh EH259RG, UK; 3 UMR-Institut des Sciences de l’Evolution de Montpellier, Centre National de la Recherche Scientifique, Institut de Recherche, Pour le Développement, Ecole Pratique des Hautes Etudes, University of Montpellier, 34295 Montpellier, France

**Keywords:** *Solea senegalensis*, flatfish, reproduction, methylation, transcriptome

## Abstract

Fish exhibit diverse mechanisms of sex differentiation and determination, shaped by both external and internal influences, often regulated by distinct DNA methylation patterns responding to environmental changes. In *S. senegalensis* aquaculture, reproductive issues in captivity pose significant challenges, particularly the lack of fertilization capabilities in captive-bred males, hindering genetic improvement measures. This study analyzed the methylation patterns and transcriptomic profiles in gonadal tissue DNA from groups differing in rearing conditions and sexual maturity stages. RRBS (Reduced Representation Bisulfite Sequencing) was employed to detect notable methylation variations across groups, while RNA was extracted and sequenced for differential expression analysis. Our findings suggest that DNA methylation significantly regulates gene expression, acting as a mechanism that can both repress and enhance gene expression depending on the genomic context. The complexity of this epigenetic mechanism is evident from the varying levels of methylation and correlation rates observed in different CpGs neighboring specific genes linked to reproduction. Differential methylation comparisons revealed the highest number of differently methylated CpGs between maturation stages, followed by rearing conditions, and lastly between sexes. These findings underscore the crucial role of methylation in regulating gene expression and its potential role in sex differentiation, highlighting the complex interplay between epigenetic modifications and gene expression.

## 1. Introduction

Understanding reproduction is crucial for the commercial production of species. Sex determination and differentiation are vital processes in most complex organisms, with mechanisms that vary widely. Teleost fish, which boast an unparalleled diversity of species, exemplify this variation. They display all known vertebrate sex determination systems and even show differences among closely related species. These mechanisms include genetic sex determination, where sex is determined by inherited genetic factors, and environmental sex determination, where factors such as temperature, pH levels, population density, and social interactions can influence sex determination and differentiation. Additionally, some species exhibit interactions between both mechanisms [1,2,3]. Therefore, identifying and characterizing the genes and pathways involved in reproduction is a fundamental initial step.

Flatfishes are a notable example of this diversity. They undergo a significant transformation from their bilateral larval stage to their adult and characteristic flat shape adapted to a demersal lifestyle. This adaptation has led to rapid diversification and a higher rate of molecular evolution compared to their bilateral counterparts. As a result, flatfish exhibit a wide variety of sex determination mechanisms, involving different master genes and unique sex determination regions [4,5].

*Solea senegalensis* has led the significant expansion and investment in the European aquaculture industry in recent years. This flatfish species boasts a high growth rate, along with a high market value and strong demand. Its production is primarily concentrated in Spain, Iceland, and Portugal, with a continuous yearly growth [6,7].

The primary challenges in *S. senegalensis* aquaculture production, aside from optimizing nutrition, controlling disease, and addressing morphological abnormalities, include reproductive issues in captivity. Specifically, captive-bred males lack fertilization capabilities. F1 males do not exhibit the complex species-specific reproductive behaviors necessary for successful spawning, such as prespawning chasing or synchronized paired spawning. Additionally, F1 males have a reduced fertilization capacity due to their low sperm volume, which hinders the implementation of genetic improvement measures through selective breeding [8,9,10,11].

Gonadal development and gametogenesis research in flatfish has primarily focused on a few species, notably *Cynoglossus semilaevis*, *Scophthalmus maximus*, and *Paralichthys olivaceus*. Numerous studies have aimed to identify and characterize genes homologous to sex-determining pathway genes in model species using mass sequencing technologies [12,13,14]. Genomic research on *S. senegalensis* includes studies analyzing transcriptomes from different tissues and culture conditions [15,16], the development of an integrated genetic map suggesting the evolution of several chromosomes in *S. senegalensis* as the outcome of Robertsonian fusions, pericentric inversions, and chromosomal rearrangements [17], and two recent chromosome-level genome assemblies providing evidence of the role of the *fshr* gene in sex determination [18,19].

Preceding research on *S. senegalensis* has investigated how temperature influences the epigenetic regulation of muscle development. This is particularly relevant due to the interest in cultivating larger female populations for aquaculture purposes. Given the interest in cultivating larger female populations, a previous study considered how temperature affects the sex ratio in Senegalese sole production. When larvae were exposed to daily temperatures of around 22 °C and nighttime temperatures of approximately 19 °C from 1 to 97 days after hatching, 70% of the population became female, with increased estradiol levels [20].

Methylation analysis of the testicular tissue in this species, focusing on genes related to reproductive traits such as sex determination and spermatogenesis, revealed greater methylation levels in F1 individuals than wild fish, and in immature wild fish compared to mature ones. These differences, particularly in genes linked to sexual differentiation, suggest a molecular basis for the behavioral differences observed between different-origin individuals, such as the absence of courtship in F1 males. The distinct cultivation conditions, with constant temperatures for F1 individuals and fluctuating temperatures for wild ones, likely contribute to these findings [21].

In this context, the quality of sperm can be significantly influenced by the methylation status of sperm DNA, and changes in DNA methylation have been linked to male infertility [12,22]. In aquaculture, fish phenotypes are influenced by a variety of external and internal factors, including temperature, salinity, population density, nutrients, and contaminants. These factors often exert their effects through specific DNA methylation patterns.

Temporary solutions have been developed to enhance the reproductive status, including artificial fertilization and nutritional strategies, as well cryopreservation protocols [23,24,25]. Although hormonal induction in F1 sole has achieved some success [26,27], the results are not yet optimal as a solution to these reproductive issues. Despite extensive research, transcriptome data for flatfish species like *S. senegalensis* remain limited. Many questions about the specific pathways and genes involved in sex determination and differentiation remain unanswered, highlighting the need for further research. Additionally, the reproductive challenges faced by hatchery-born individuals complicate the implementation of precise aquaculture practices for this species.

Epigenetic changes, particularly DNA methylation, are crucial for how organisms adapt to environmental changes, influencing gene expression through the conversion of cytosines to 5′-methylcytosine by DNA methyltransferases (DNMTs). The patterns of DNA methylation are tissue-specific. Although DNA methylation is primarily found in CpG dinucleotides, these modifications can also occur at CpG, CHG, or CHH regions (where H represents A, T, or C). DNMTs facilitate methylation in these areas, while Ten-Eleven Translocation (TET) methylcytosine dioxygenases (TET1, TET2, and TET3) aid in the demethylation of the methylated cytosines [28,29,30].

These modifications influence various genetic processes, including genome stability, genomic imprinting, and transcription. While traditionally linked to gene repression, DNA methylation can also enhance gene expression depending on its location. Decreased expression has been associated with hypermethylation of CpG sites in the promoter and gene body regions. Conversely, increased expression has been linked to hypermethylation of CpG sites downstream of the gene body [31,32].

The impact of epigenetics on fish sex determination has been extensively described. Modifications in DNA methylation have been associated with temperature-induced sex differentiation and environmental sex differentiation regulation. However, while epigenetics holds great promise for improving aquaculture, practical applications remain scarce [33].

The primary aim of this study was to examine the variations in methylation patterns within the gonadal tissue (ovaries and testes) of Senegalese sole and to explore how methylation functions as an epigenetic mechanism in gene expression regulation at two different stages of maturation, under both wild and captive breeding conditions. Our results revealed significant methylation differences between sexes and rearing groups, which were also observed in gene expression comparisons. Additionally, the integration of methylome and transcriptome data showed that methylation acts as both a suppressor and enhancer of gene expression.

These findings underscore the crucial role of methylation in regulating gene expression through maturation and its potential role in sex differentiation, highlighting the complex interplay between epigenetic modifications and gene expression in this species.

## 2. Materials and Methods

### 2.1. Gonadal Tissue Sampling

Individuals were collected from two groups: one that was artificially bred (F1) at IFAPA and wild fish from the Cádiz, Andalusia. The samples were categorized based on three criteria: gender, origin, and maturity level.

Sampling occurred during the spawning season (May–June), with immature individuals collected year-round. Gonadal tissue samples were taken from 24 *S. senegalensis* specimens. This study included eight groups based on the previous criteria: immature wild (IW), mature wild (MW), immature F1 (IF), and mature F1 (MF). We examined 6 adult males (4 wild, 2 F1), 6 adult females (2 wild, 4 F1), 6 immature males (4 wild juveniles, 2 F1 juveniles), and 6 immature females (3 wild juveniles, 3 F1 juveniles). Wild fish were collected using trawl nets in coastal areas. Cultured fish sample sizes were limited due to availability and high early-stage mortality rates.

**Figure A1:**
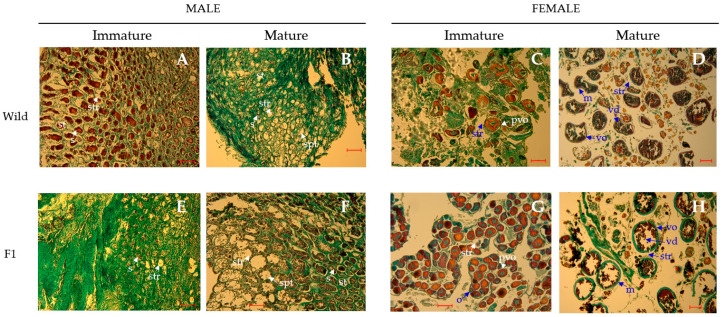
Histological analysis of gonadal maturation stages in *Solea senegalensis*. (**A**) IWM: Immature Wild Male, (**B**) MWM: Mature Wild Male, (**C**) IWF: Immature Wild Female, (**D**) MWF: Mature Wild Female, (**E**) IFM: Immature F1 Male, (**F**) MFM: Mature F1 Male, (**G**) IFF: Immature F1 Female, and (**H**) MFF: Mature F1 Female. Notes: Meaning of abbreviations, spt: spermatozoa, st: seminiferous tubule, s: spermatogonia, str: stroma, o: oogonia (germ cell), pvo: pre-vitellogenic oocyte, vo: vitellogenic oocyte (cell in maturation phase), vd: vitello drops, and m: membrane (formed by the theca, granulosa cells, and pellucid membrane). Scale bar in (**A**–**C**,**E**–**G**) (10×): 50 µm; and scale bar in (**D**) and (**H**) (4×): 100 µm.

Size and weight were used as indicators of sexual maturation. Mature specimens were obtained during spawning months, and their maturity was confirmed by performing gonadal tissue dissections; a portion of each sample was used for histological analysis to verify the maturation stages (Figure A1). The histological process involved standard procedures: fixation in formalin, dehydration through a graded ethanol series, paraffin embedding, microtome sectioning, and staining with hematoxylin and eosin.

F1 specimens reached sexual maturity under stable conditions. Factors like feeding patterns, population density, and oxygen levels can serve as epigenetic modulators. Even after reaching sexual maturity, F1 individuals encountered reproductive difficulties, such as lack of reproductive behavior. The fish were transferred to the laboratory in oxygenated containers and anesthetized with clove oil (90–100 mg/L). Both wild and cultured fish were maintained in oxygenated containers for 1 to 2 h before necropsy to reduce stress and ensure optimal conditions. Gonad samples were stored in RNAlater^®^ TissueProtect at −80 °C until DNA extraction. The experimental protocol adhered to EU guidelines (86/609/EU) and received approval from the Ethics Committee of the University of Cadiz, Spain.

### 2.2. Gonadal DNA and RNA Samples’ Extraction

DNA was extracted from testicular tissue using the DNeasy Blood & Tissue Kit (Qiagen). Following extraction, the DNA samples underwent purification and size selection using the HighPrep™ PCR Clean-up System (MagBio Genomics Inc., Gaithersburg, MD, USA). A NanoDrop™ 2000C spectrophotometer (Thermo Fisher Scientific, Waltham, MA, USA) was used for quality assessment, while concentration was measured using a Qubit 4 fluorometer (Thermo Fisher Scientific, Waltham, MA, USA).

RNA extraction was performed from 0.5 mg of gonadal tissue using TRIzol™ Reagent (Thermo Fisher Scientific, Waltham, MA, USA), following the manufacturer’s protocol. Quantification and purity assessment of the extracted RNA were conducted via fluorometry using the Qubit system (Invitrogen, Thermo Fisher Scientific, Waltham, MA, USA). RNA integrity (RIN) was determined using a Bioanalyzer Agilent 2100, following the manufacturer’s protocol. Samples with an RIN greater than 4 were selected for transcriptome sequencing.

### 2.3. RRBS and RNA Libraries’ Preparation and Sequencing

For RRBS library preparation, genomic DNA (100 ng per sample) was digested with MspI for 12 h using the Diagenode Premium Kit (Hologic Diagenode, Marlborough, MA, USA). Following end preparation, adaptor ligation, and size selection, sample concentrations were measured using qPCR. Samples with similar concentrations were pooled in groups of six and subjected to bisulfite conversion, including both methylated and unmethylated control DNA. RRBS libraries were enriched by PCR, purified with AMPure XP Beads, and assessed for fragment size distribution using an Agilent 2200 Bioanalyzer (© Agilent Technologies, Inc., Santa Clara, CA, USA). Libraries were measured with a Qubit fluorometer, pooled, and sequenced (150 bp paired end) on the Novaseq Illumina platform by Novogene’s genome sequencing service.

For mRNA library preparation, mRNA was purified from total RNA using poly-T oligo-attached magnetic beads. After fragmentation, the first strand of cDNA was synthesized with random hexamer primers, followed by the second-strand synthesis. Subsequent steps included end repair, A-tailing, adapter ligation, size selection, amplification, and purification. RNA libraries were quantified using Qubit and real-time PCR, and their size distribution was assessed with a bioanalyzer. Samples with an RNA integrity number greater than 6 were selected for transcriptome sequencing. Libraries were then pooled and sequenced on Novogene’s Illumina platforms based on effective library concentration and data requirements.

### 2.4. Quality Control and Data Processing

We used Trim Galore 0.6.61 to remove Illumina sequencing adapters, low-quality base calls (Phred quality score < 20), and short reads (<20 bp). To ensure the quality of the fastq file data, we employed FastQC 0.12.0.

We performed RRBS alignment, through an in silico bisulfite conversion of the reference genome following the last version of the *S. senegalensis* published genome (GenBank assembly GCA_919967415.2 from NCBI). The RRBS sequence reads’ alignment to the in silico converted *S. senegalensis* reference genome, as well as the subsequent methylation analysis and methylation calling of each cytosine, were performed using Bismark 0.22.3 for CpG coverage analysis.

RNA paired-end reads were aligned to the genome using Hisat2 v2.0.5. Feature Counts v1.5.0-p3 was employed to count the number of reads mapping to each gene and to calculate the FPKM (Fragments Per Kilobase Million) of each gene.

### 2.5. Differential Analysis

For differential methylation analysis, coverage output files were processed according to Mukiibi et al. (2022) using the Bioconductor edgeR 3.42.2 R package [34]. CpG sites that were consistently methylated, unmethylated, or had low coverage (fewer than 8 reads per sample) were excluded. To explore methylation patterns, multidimensional scaling (MDS) analysis was performed using the R prcomp function. CpG read counts within sample libraries were normalized to the average library size for consistency. Likelihood ratio tests were used to compare methylation rates between different study groups. CpG sites with significant differential methylation were identified using a false discovery rate threshold of 0.01 (Benjamini–Hochberg correction).

Using categories based on the criteria for sample collection (sex, maturity, and origin), 12 comparisons were conducted for both differential methylation analysis and transcriptomic profile comparisons between groups. These comparisons aimed to examine the potential effects of those factors on methylation and gene expression. The following group comparisons were performed: Mature F1 Male vs. Mature Wild Male (MFM/MWM), Immature F1 Male vs. Immature Wild Male (IFM/IWM), Immature Wild Male vs. Mature Wild Male (IWM/MWM), Immature F1 Male Vs. Mature F1 Male (IFM/MFM), Mature F1 Female vs. Mature Wild Female (MFF/MWF), Immature F1 Female vs. Immature Wild Female (IFF/IWF), Immature Wild Female vs. Mature Wild Female (IWF/MWF), Immature F1 Female vs. Mature F1 Female (IFF/MFF), Mature F1 Female vs. Mature F1 Male (MFF/MFM), Immature F1 Female vs. Immature F1 Male (IFF/IFM), Mature Wild Female vs. Mature Wild Male (MWF/MWM), and Immature Wild Female vs. Immature Wild Male (IWF/IWM).

Methylation differences were visualized on heatmaps and volcano plots generated with the R package gplots v3.1.1. Functional annotation was performed based on the Senegalese sole annotation file (GenBank assembly GCA_919967415.2 from NCBI), using HOMER software v4.1, indicating CpG site positions in relation to gene promoters, introns, exons, and transcription start and termination sites.

The R package DESeq2 (1.20.0) was employed for differential expression analysis between two conditions/groups. We analyzed the correlation of gene expression levels between samples using the FPKM values of all genes in each sample and calculated the correlation coefficients within and between groups. These coefficients were visualized in heatmaps to display the differences between groups and the consistency within groups. For differential expression analysis with two biological replicates per condition, Principal Component Analysis (PCA), volcano plots, and heatmaps were also generated using the ggplot2 package in R (Version 3.0.3) within Novogene’s NovoMagic Tool (https://eu-magic.novogene.com/, accessed on 1 November 2024).

To integrate methylation and transcription data, a correlation analysis was conducted to identify significant associations (*p*-value < 0.05) between methylation levels and gene expression. Differentially methylated regions and differentially expressed genes (DEGs) were identified and compared to find overlapping genes. Pearson’s correlations between the percentage of DNA methylation at CpG sites and the counts per million (cpm) of their overlapping genes were computed.

Due to the difficulty in obtaining specimens, we were unable to acquire the desired number of samples. Consequently, our farmed juvenile and adult sample size was restricted, potentially reducing certain variability. Specifically, we could not achieve more than two F1 replicates for the male groups due to their short supply, which may have introduced some bias into the analysis. Furthermore, the mature wild female group lacked a third replicate. Despite these limitations, we believe the experiment remained scientifically valuable for studying *S. senegalensis*. However, it is important to consider these constraints in the analysis and interpretation of the results. Further studies with larger sample sizes are necessary to validate and expand upon these findings.

### 2.6. GO and KEGG Enrichment Analysis

The clusterProfiler R package was used to perform Gene Ontology (GO) and KEGG enrichment analyses on differentially methylated and expressed genes using Novogene’s Novomagic Platform (https://eu-magic.novogene.com/, accessed on 1 November 2024).

GO terms with a corrected *p*-value below 0.05 were deemed significantly enriched by these genes. Additionally, the clusterProfiler R package was used to test the statistical enrichment of differentially expressed genes in KEGG pathways. Associations with GO and KEGG terms were visualized in bar and scatter plots.

## 3. Results

### 3.1. Data Quality Control of Sequencing Information

RRBS libraries from gonadal tissue sequencing yielded a total of 2,180,046,558 raw paired-end reads (n = 12). The Q20 and Q30 values ranged from 93.04 ± 1.17% to 85.46 ± 1.47% (Mean ± S.D), respectively, across samples (Table A1). After filtering, 2,133,878,766 reads remained. The mapping efficiency was 62.33 ± 4.54%. Following quality control, a total of 2,623,861,381 cytosines in the CpG context were reported, showing some level of methylation, which accounted for 53.63 ± 0.20% of the total number of analyzed cytosines (Table A2).

RNA sequencing of gonadal samples yielded a total of 1,239,301,554 clean reads. The Q20 and Q30 values ranged from 97.52 ± 0.28% to 92.99 ± 0.60%, respectively, across samples (Table A1). On average, 88.57 ± 1.00% of the sequences aligned with the *S. senegalensis* reference genome; the mapping efficiency was 94.60 ± 0.01%. Among the mapped reads, 82.48 ± 4.88% corresponded to exons, 5.94 ± 1.94% to introns, and 11.57 ± 3.20% to intergenic regions. Overall, these results indicate that the sequencing experiment was successful and yielded high-quality data.

### 3.2. Methylation Patterns’ Study and Differential Analysis

**Figure 1:**
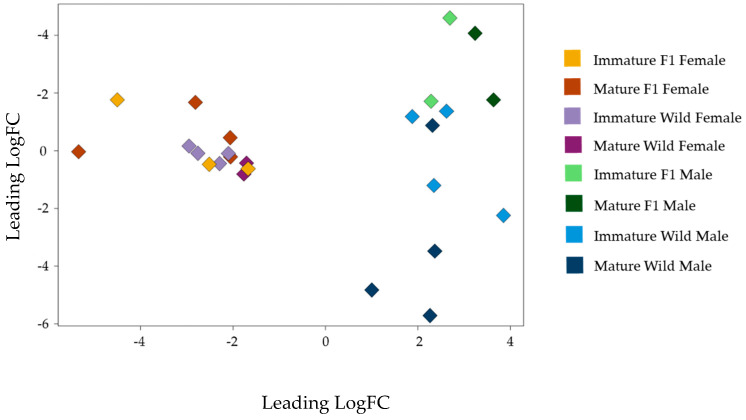
Multidimensional scaling (MDS) plot for methylation profiles among *S. senegalensis* samples (created using the edgeR package). Notes: The variations in methylation profiles among *S. senegalensis* samples are shown for the eight study groups—mature wild males and females, immature wild males and females, mature F1 males and females, and immature F1 males and females. Groups are each depicted in distinct colors. Individual samples are represented by colored tags, with each corresponding to its respective group. The proximity reflects the similarity among samples within groups.

A general multidimensional scaling plot of global methylation levels reveals primary differences among males and females. Within each sex, F1 and wild groups can be distinguished, particularly among male samples, with F1 males exhibiting the most significant methylation levels. Independent comparisons of males and females also revealed differences between the F1 and wild groups. Notably, significant differences were observed between groups of different maturity (immature and wild) in both sexes (Figure 1).

**Figure 2:**
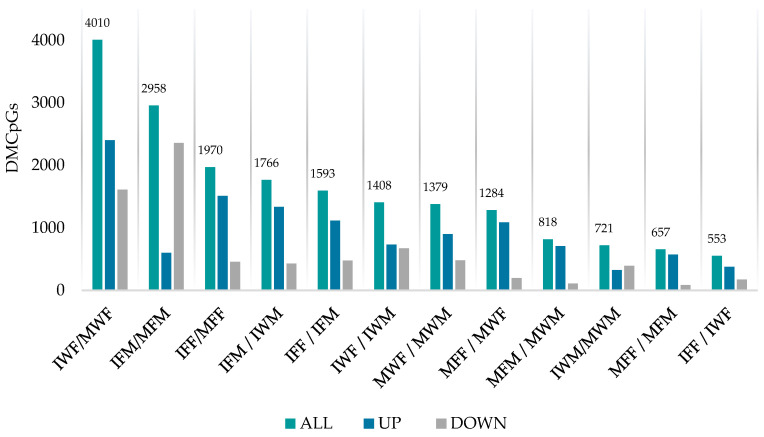
Total number of DMCpGs (y-axis) across group comparisons (x-axis). Group comparison keys are shown as follows: Mature F1 Male vs. Mature Wild Male (MFM/MWM), Immature F1 Male vs. Immature Wild Male (IFM/IWM), Immature Wild Male vs. Mature Wild Male (IWM/MWM), Immature vs. Mature F1 Male (IFM/MFM), Mature F1 Female vs. Mature Wild Female (MFF/MWF), Immature F1 Female vs. Immature Wild Female (IFF/IWF), Immature Wild Female vs. Mature Wild Female (IWF/MWF), Immature F1 Female vs. Mature F1 Female (IFF/MFF), Mature F1 Female vs. Mature F1 Male (MFF/MFM), Immature F1 Female vs. Immature F1 Male (IFF/IFM), Mature Wild Female vs. Mature Wild Male (MWF/MWM), and Immature Wild Female vs. Immature Wild Male (IWF/IWM).

Taking into account the study groups, we conducted 12 comparisons of differential methylation profiles (Table A4). Only CpG sites with adequate coverage (>8 counts) and variation in methylation levels across samples, with a false discovery rate below 0.01 adjusted using the Benjamini–Hochberg method, were considered significantly differentially methylated. The analysis revealed a total of 19,117 CpG sites with significant differential methylation across the performed comparisons (Figure 2), which were considered for the genetic annotation step.

Generally, in terms of differently methylated CpGs (DMCpGs), the comparisons revealed that the largest number of DMCpGs were observed between different maturation states, followed by comparisons between different rearing groups, and lastly between sexes.

The overall mean methylation difference, measured as log2 fold change (logFC), was 1.50 ± 0.20. While the average fold change values remained close among comparisons (ranging from 1.04 ± 0.22 to 1.75 ± 0.31) the highest differences among the studied groups were observed when comparing groups of different maturity, followed by comparisons between different sex groups, and finally groups differentiated by their rearing.

Focusing on the analysis of male groups, among those showing a marked reproductive dysfunction, generally a larger number of up-methylated CpGs were observed in F1 males when compared to wild males, and in immature individuals compared to their mature counterparts. This trend was also observed in females apart from the comparison between immature and mature F1 female groups, where the mature group exhibited a larger number of DMCpGs. When comparing groups of males and females, the latter consistently exhibited higher levels of methylation. However, an exception was observed in the immature F1 groups, where males showed a larger number of up-methylated CpGs.

**Figure 3:**
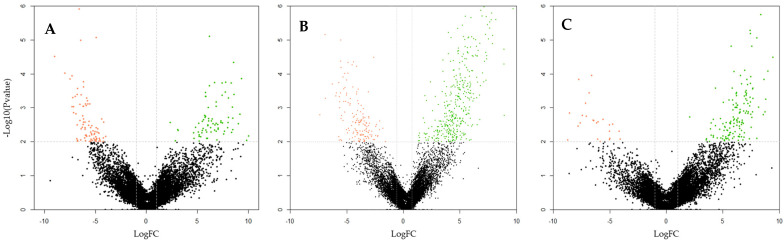
Volcano plots for methylation differences between *S. senegalensis* groups: (**A**) F1 vs. Wild; (**B**) Females vs. Males; (**C**) Immature vs. Mature. Notes: The volcano plots were generated using the R package gplots v3.1.1. The x-axis represents the fold change value, while the y-axis shows the *p*-value. Dots representing samples that exceed the established significance limits are colored green (up-methylated) and red (down-methylated).

When classifying samples into specific categories, they were grouped to compare the general categories of sex, maturity, and origin. This approach allowed for the identification of methylation differences between these broad categories within the total number of individuals. Consequently, comparisons were made between males and females, mature and immature individuals, and F1 and wild individuals. Significant differences were observed in all comparisons, with higher methylation levels in females, immature individuals, and F1 individuals when compared to their respective counterparts (Figure 3).

Despite our efforts to include more than three samples in each studied group, the limited number of F1 samples allowed us to include only two replicates for the immature and mature F1 male groups, which might have caused some bias in the analysis. Additionally, a third replicate in the mature wild female group was missing. However, RRBS offers extensive coverage of CpG-rich regions in the genome, which can often offset the absence of biological replicates [35].

The high-resolution data from RRBS can yield robust and reliable results, even with a limited number of samples. Despite its limitations, our study provides valuable insights and we hope it will inspire future research with larger sample sizes.

### 3.3. Genomic Annotation Based on Differentially Methylated Regions

The reported DMCpG sites were identified and annotated according to their locus and proximity to genes, following the annotation of the Senegalese sole genome (GenBank assembly GCA_919967415.2 from NCBI), and further categorized based on their location regarding the nearest gene, as follows: gene promoters/transcription start sites (TSS, ±1 kbp), introns, exons, transcription termination sites (TTS, ±1 kbp), or intergenic regions.

These DMCpGs were annotated to 4581 different genes. Most DMCpGs were found in intronic and intergenic regions. The distribution was as follows: 8.00% in promoters/transcription start sites (TSS, ±1 kbp), 15.15% in exons, 40.45% in introns, 31.53% in intergenic regions, and 4.87% in transcription termination sites (TTS, ±1 kbp). Additionally, 13.08% were in first intron regions.

Upon annotation, we found that the identified DMCpG sites were located nearby or within various *sox* family transcription factors, genes related to the steroidogenic pathway (*dmrt2ab*, *hsd3b7*), and genes involved in methylation and demethylation processes (*tet3*, *kdm1b*, *kdm5b*, *kdm6ba*).

Our findings revealed CpG methylation differences among certain groups linked to genes that play a role in sex determination and reproduction, gonadal differentiation and sperm production, and germ cell regulation and development (*fshr*, *dmrt*, *sox9a*, *bcar1*, *hsd17b1*, *hox*).

Methylation changes in intronic and intergenic regions in these areas could significantly influence gene expression, while the presence of DMCpGs in promoters and transcription start sites (TSSs) as well as exons suggests potential direct impacts on gene transcription. Additionally, a GO enrichment analysis of the annotated genes highlighted several enriched regulatory processes, such as the regulation of signaling (GO:0023051) and response to stimuli (GO:0050896), particularly in the mature F1/Wild males comparison. These findings are especially relevant given the challenges associated with farming this species.

**Figure 4:**
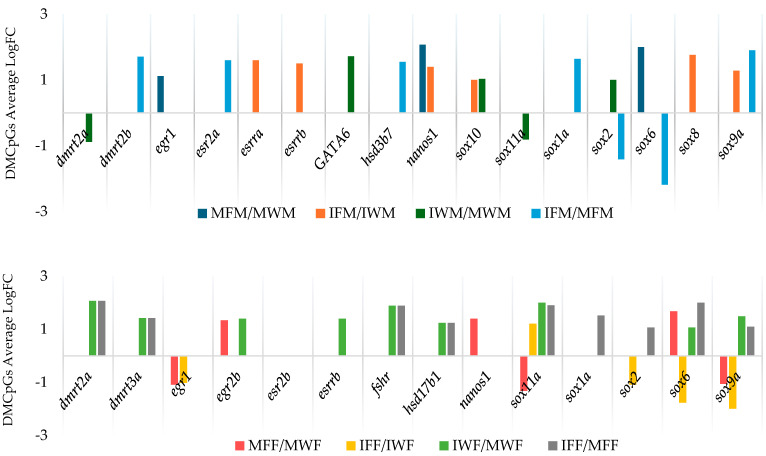
Average methylation differences (mean LogFC) in gene-associated DMCpGs among female groups of study in *Solea senegalensis.* Notes: Group comparison keys are shown as follows: MFM/MWM (Mature F1 vs. Wild Male), IFM/IWM (Immature F1 vs. Wild Male), IWM/MWM (Immature vs. Mature Wild Male), IFM/MFM (Immature vs. Mature F1 Male), MFF/MWF (Mature F1 Female vs. Mature Wild Female), IFF/IWF (Immature F1 Female vs. Immature Wild Female), IWF/MWF (Immature Wild Female vs. Mature Wild Female), IFF/MFF (Immature F1 Female vs. Mature F1 Female).

In the differential expression analysis, we focused on specific genes related to reproduction [36,37] identified through DMCpGs’ annotation, with special attention paid to males of different origins (Figure 4), to study whether they exhibited differential expression. Some of the genes related to reproduction, such as those in the *sox* transcription factor family and the estrogen receptor genes, had differentially methylated CpG sites (DMCpGs) in their vicinity according to gene annotation. However, these genes did not exhibit expression differences that could be directly associated with the observed methylation differences. This suggests that methylation can act as a regulatory mechanism influencing gene expression over time or under specific conditions; yet, while methylation changes are present, they may not always translate into immediate changes in gene expression [38].

When we compared mature F1 and wild groups, *egr1* and *nanos1* exhibited greater methylation in the mature F1 group compared to its wild counterpart and were also down-regulated (LogFC > −2). However, when comparing immature F1 and wild-type groups, despite several genes showing methylation differences between the groups, no significant differences in the expression of these genes were observed.

When comparing immature and mature wild groups, *GATA6* showed greater methylation in immature males, though it was down-regulated compared to their mature counterparts (LogFC = −1.40). Conversely, *dmrt2a* showed lower methylation levels and was up-regulated in the immature group compared to its mature counterparts (LogFC = 1.92).

Lastly, in the immature and mature F1 group comparison, *dmrt*2b, *esr2a*, *hsd3b7*, and *sox1a* showed greater methylation levels and lower expression in the immature group compared to their mature counterparts, although the expression differences were less significant (LogFC < 1.5).

### 3.4. Outline of Transcriptome Profiles and Differential Expression Analysis

**Figure 5:**
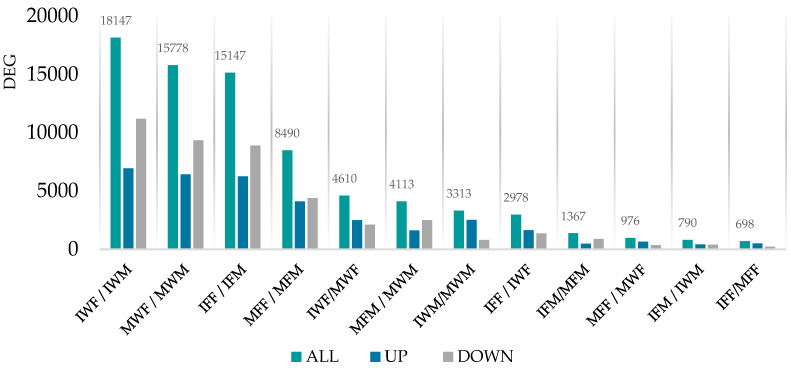
Counts for differentially expressed genes (y-axis) across pairwise comparisons (x-axis) in *Solea senegalensis.* Notes: Group comparison keys are shown as follows: MFM/MWM (Mature F1 Male vs. Mature Wild Male), IFM/IWM (Immature F1 Male vs. Immature Wild Male), IWM/MWM (Immature Wild Male vs. Mature Wild Male), IFM/MFM (Immature F1 Male vs. Mature F1 Male), MFF/MWF (Mature F1 Female vs. Mature Wild Female), IFF/IWF (Immature F1 Female vs. Immature Wild Female), IWF/MWF (Immature Wild Female vs. Mature Wild Female), IFF/MFF (Immature F1 Female vs. Mature F1 Female), MFF/MFM (Mature F1 Female vs. Mature F1 Male), IFF/IFM (Immature F1 Female vs. Immature F1 Male), MWF/MWM (Mature Wild Female vs. Mature Wild Male), and IWF/IWM (Immature Wild Female vs. Immature Wild Male).

**Figure A2:**
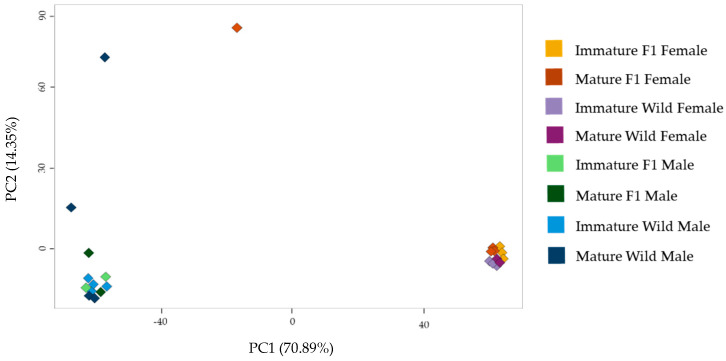
Principal Component Analysis (PCA) plot based on gene expression profiles among *S. senegalensis* samples (generated using Novogene’s Novomagic Platform). Notes: PC1 (Principal Component 1) and PC2 (Principal Component 2) show the amount of variability in the data in different directions, representing distinct gene expression profiles. The variations in expression profiles among *S. senegalensis* samples are shown for the eight study groups—mature wild males and females, immature wild males and females, mature F1 males and females, and immature F1 males and females. Groups are each depicted in distinct colors. Individual samples are represented by colored tags, with each corresponding to its respective group. The closeness indicates the degree of similarity among the *S. senegalensis* samples within each group.

A Principal Component Analysis (PCA) of the overall samples revealed significant differences between the sexes. The analysis showed that the samples formed two distinct clusters, one for each sex, indicating a clear sexual dimorphism (Figure A2). Additionally, the PCA was conducted independently for each sex, considering groups of different origins and maturities. When only samples of the same sex were considered, it was possible to differentiate the distributions among those of different origins, with a better grouping of those that shared the same upbringing. Within each sex, F1 and wild groups were distinguishable, particularly among female samples. These differences were also reflected in the differential gene expression analysis (Figure 5).

The comparisons between samples of different sexes revealed the highest number of differentially expressed genes (DEGs). This was followed by differences in maturation status, and lastly, by differences between wild-type and F1 individuals.

**Figure 6:**
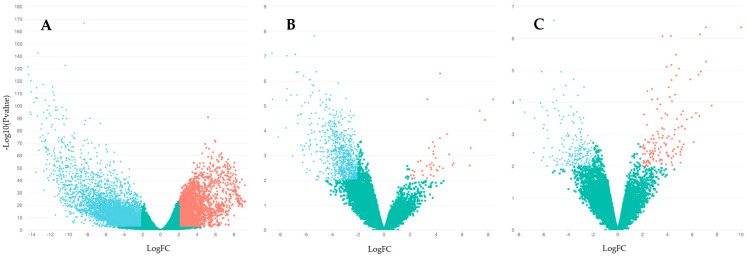
Volcano plots for differentially expressed genes between *S. senegalensis* study groups: (**A**) Females vs. Males; (**B**) Immature vs. Mature; (**C**) F1 vs. Wild. Notes: The volcano plots were generated using the ggplot package in R Version 3.03 on Novogene’s Novomagic Plaform. The x-axis represents the fold change value, while the y-axis shows the *p*-value. Dots representing samples that exceed the established significance limits are colored red for up-regulated and blue for down-regulated, the green dots indicate values that fall below the established significance thresholds..

To identify expression differences across general categories, samples were grouped to compare the broader categories of sex, maturity, and origin. Within the total number of individuals, comparisons were made between males and females, mature and immature individuals, and F1 and wild individuals. Significant expression differences were observed in all comparisons, with a larger number of down-regulated genes in males, mature individuals, and wild-type specimens compared to their respective counterparts (Figure 6).

### 3.5. Analysis of Differential Gene Expression and Patterns in Males

A comparative analysis of gene expression patterns in males showed notable differences among the groups. In the comparison between mature males with different origins, 154 genes were up-regulated in mature F1 males, while 374 genes were down-regulated in mature wild males, highlighting differences between these groups.

When comparing immature to mature wild males, 129 genes were up-regulated in immature wild males, while 1528 genes were down-regulated in mature wild males, indicating a significant shift in gene expression during maturation.

Similarly, when comparing immature F1 and wild males, 169 genes were up-regulated in immature F1 males, and 56 genes were down-regulated in immature wild males, suggesting higher gene expression in F1 males.

Lastly, the comparison between immature and mature F1 males showed 302 genes up-regulated in immature F1 males, and 179 genes down-regulated in mature F1 males, indicating significant changes in gene expression during maturation.

Wild males exhibited a larger shift in gene expression during maturation when compared to F1 males. Additionally, F1 males, both mature and immature, tended to show greater expression in certain genes compared to their wild counterparts. Overall, these differences suggest that both maturation and origin significantly influence gene expression patterns.

### 3.6. Analysis of Differential Gene Expression and Patterns in Females

A similar trend as that observed in male specimens was also observed in females. Specifically, in the comparison between mature wild and F1 females, 161 genes were up-regulated in mature F1 females, while 62 genes were down-regulated in mature wild females.

When comparing immature and mature wild females, 1429 genes were up-regulated in immature wild females, whereas 1830 genes were down-regulated in mature wild females.

Similarly, when comparing immature F1 and wild females, 706 genes were up-regulated in immature F1 females, and 528 genes were down-regulated in immature wild females.

Lastly, the comparison between immature and mature F1 females showed 27 genes up-regulated in immature F1 females and 889 genes down-regulated in mature F1 females.

As in males, wild females exhibit a larger shift in gene expression during maturation compared to F1 females. However, mature and immature F1 females showed a greater expression of certain genes when compared to the wild groups.

Furthermore, the comparative analysis of gene expression patterns revealed substantial differences between males and females, highlighting the significant influence of sex on gene expression. Females, both immature and mature, tended to show a higher expression of certain genes compared to their male counterparts, with more pronounced differences observed in wild groups compared to F1 groups. Additionally, the differences in gene expression between immature and mature individuals within the same sex (for both wild and F1 groups) suggest that maturation significantly influences gene expression, with sex-specific changes.

### 3.7. Annotation and Enrichment Analysis of Differentially Expressed Genes

**Figure A3:**
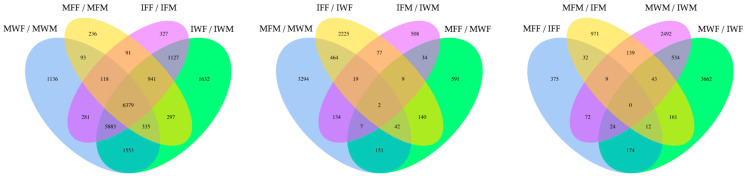
Differential expression Venn diagrams illustrating the differential genes of the compared groups in *Solea senegalensis*. Notes: The sum of all the numbers in the circle represents the total number in the compared groups, and the overlapping area indicates the number of differential genes shared between the groups in different comparison sets. Group comparison keys are shown as follows: MFM/MWM (Mature F1 Male vs. Mature Wild Male), IFM/IWM (Immature F1 Male vs. Immature Wild Male), IWM/MWM (Immature Wild Male vs. Mature Wild Male), IFM/MFM (Immature F1 Male vs. Mature F1 Male), MFF/MWF (Mature F1 Female vs. Mature Wild Female), IFF/IWF (Immature F1 Female vs. Immature Wild Female), IWF/MWF (Immature Wild Female vs. Mature Wild Female), IFF/MFF (Immature F1 Female vs. Mature F1 Female), MFF/MFM (Mature F1 Female vs. Mature F1 Male), IFF/IFM (Immature F1 Female vs. Immature F1 Male), MWF/MWM (Mature Wild Female vs. Mature Wild Male), and IWF/IWM (Immature Wild Female vs. Immature Wild Male).

Following the gene expression level analysis, a total of 26,174 genes were annotated. The GO enrichment analysis revealed 1008 enriched GO terms, including those related to reproduction and reproductive processes (GO:0000003, GO:0022414). As previously described, 12 comparisons were conducted among groups (Figure A3), all of which showed significant differences.

**Figure 7:**
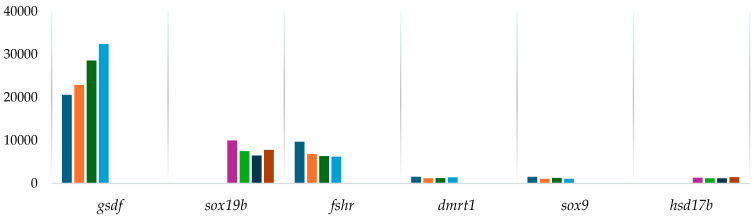

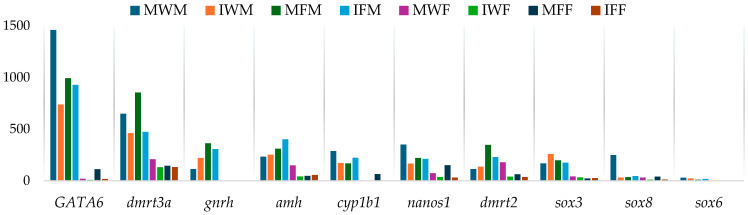
Bars graph representation of gene expression levels measured as gene counts for reproduction-related genes across studied groups in *Solea senegalensis.* Notes: Group keys are shown as follows: MWM (Mature Wild Male), IWM (Immature Wild Male), MFF (Mature F1 Female), IWF (Immature Wild Female), IFF (Immature F1 Female), IFM (Immature F1 Male), MFM (Mature F1 Male), and MWF (Mature Wild Female).

The results indicate that gene expression varies significantly between sexes and developmental stages, particularly in genes related to reproduction and sex differentiation (Figure 7 and Table A3). Genes such as *gsdf*, *fshr*, and *amh* exhibited higher expression levels in both immature and mature male groups compared to female groups, underscoring their crucial roles in male reproductive functions. This pattern was further seen in several transcription factors showing an elevated expression in males, particularly in mature wild and F1 males. Conversely, *sox19b* and *hsd17b* genes were more prominently expressed in females, indicating their involvement in female reproductive processes.

### 3.8. Integration of Methylation and Expression Data

We assessed the correlation between methylation levels and gene expression to study methylation’s potential impact on transcriptional regulation. We focused on mature male sole groups to understand the relationship between DNA methylation and gene expression. By analyzing the methylation percentage in the reported cytosines from the coverage files and the expression levels as counts per million (cpm) of the annotated genes in the RNA-seq data, we aimed to investigate the potential repression of gene expression due to increased methylation.

Out of 74,523 CpG sites with variable methylation, 3359 sites showed a significant correlation (*p*-value < 0.05) with the expression of neighboring genes. The mean correlation values remained consistent across different contexts (intro, exon, intergenic, promoter-TSS regions, TTS region), with a mean correlation value of |0.5 ± 0.28|; 25% of CpGs exceeded a |0.7| correlation, while 32% ranged between |0.4| and |0.5|.

Among the intronic CpGs, 48% showed a negative correlation, followed by intergenic (23%), exon (18%), promoter-TSS (9%), and TTS regions (2%). Overall, methylation in the putative proximal promoter-TSS regions, exons, introns, and to a lesser extent the TTS regions, exhibited the strongest negative correlations with gene expression. No significant correlation values differences were observed between first-intron/exon regions. Approximately 45% of the methylated sites negatively correlated with gene expression, while 55% showed a positive correlation (Table S1).

A Gene Ontology (GO) functional enrichment analysis was conducted on genes neighboring DMCpGs that exhibited a significant correlation between methylation and gene expression. Among the DMCpGs with a negative correlation, the most significant terms (*p*-value < 0.05) included developmental processes, transcriptional regulation, and stimulus response pathways (GO:0032502, GO:0140110, GO:0042221, GO:0009719). Additionally, calcium ion binding (GO:0005509) was significantly enriched when exclusively analyzing genes with a strong correlation (r < −0.9).

Notably, the enrichment analysis of genes neighboring DMCpGs with a positive correlation revealed fewer significantly enriched GO terms, with the most frequent being cell adhesion (GO:0007155) and regulation processes (GO:0010646, GO:0023051). Similar results were observed when analyzing genes with a strong (r > 0.9) positive correlation, including transcription regulator activity (GO:0140110) and developmental processes (GO:0032502).

Additionally, the KEGG enrichment analysis revealed significantly (*p* < 0.05) different pathways in the genes to which DMCpGs were associated, which showed a negative correlation (Figure A3), including some relevant to reproduction (MAPK, TGF-beta signaling pathways) [39,40].

## 4. Discussion

This study addresses a reported area of complexity by analyzing the methylome and gonadal transcriptome of *S. senegalensis*, identifying potential interactions of specific genes related to reproduction, and examining the effects of sex, maturity, and rearing conditions on these mechanisms.

### 4.1. RRBS Methylation Analysis of Gonadal Tissue

The sequencing of RRBS libraries from gonadal tissue revealed that over 2.6 billion cytosines in the CpG context showed methylation, accounting for about 53.63% of the total analyzed cytosines. While CpG islands, often associated with genes and their promoter regions, may exhibit different methylation patterns [41], this percentage is slightly lower than the typical 60–80% methylation observed in vertebrate genomes [42]. This discrepancy could be attributed to the species-specific genomic and epigenetic landscape, including differences in the distribution and density of CpG sites, as well as the presence of unmethylated CpG islands. Additionally, the specific tissue type (gonadal tissue) and the developmental stage of the samples analyzed could also influence the overall methylation levels. Overall, while the percentage is lower than the typical range, it remains within a biologically plausible range given these factors [43].

The mean mapping efficiency was 62%, which falls towards the lower end of the typically accepted range (>60%), likely due to biases from the bisulfite conversion process, the repetitive characteristics of CpG regions targeted by RRBS, and possible discrepancies with the reference genome [44].

The analysis of DMCpG levels revealed significant differences when comparing sex, rearing conditions, and maturity groups. Primary differences in overall methylation were observed between males and females, indicating a substantial role of sex in methylation patterns. This could be due to the specificity of ovarian and testicular gonadal tissues, among other factors [36]. Grouping samples by sex, maturity, and origin allowed for the identification of methylation differences across these broad categories, with significant differences observed in all comparisons. As further discussed, higher methylation levels were found in females, immature individuals, and F1 individuals compared to their counterparts.

Distinct methylation patterns were observed within each sex, distinguishing F1 and wild groups. F1 males exhibited the highest methylation levels, particularly those with reproductive dysfunction, who showed an increased number of up-methylated CpGs relative to wild-type males. This suggests that captivity may significantly impact methylation patterns and potentially affect reproductive abilities [21].

Previous studies suggest that changes in gene expression caused by raising salmonids in captivity can affect the fitness of the first generation of hatchery fish, highlighting the potential role of methylation after just one generation [45]. Raising organisms in captivity often results in stable conditions, with minimal fluctuations in factors like temperature, salinity, pH, and daylight. In European seabass, the role of temperature in the methylation of genes related to reproduction has been highlighted, revealing a complex epigenetic layer influenced by both the genetic background and early developmental environment, which contributes to the sexual phenotypic outcome [46].

Significant differences were found in immature individuals compared to their mature counterparts in both sexes, with females generally exhibiting higher methylation levels than males. These findings suggest a potential sex-specific role of methylation patterns influencing maturity. Similarly, in the model organism Danio rerio, sex-specific epigenetic regulation and transcription of genes involved in reproduction have been described when analyzing gonadal tissue, showing both positive and negative correlations [36].

While the limited number of F1 samples and lack of replicates must be considered, the RRBS data analysis revealed significant differences among methylation profiles. Overall, differential methylation comparisons showed the largest number of DMCpG sites between different maturation statuses, followed by rearing conditions, and finally between sexes. These findings suggest a potential role of environmental factors in shaping methylation profiles in *S. senegalensis*, which play a crucial role in maturation [47].

### 4.2. Annotation and Distribution of Differentially Methylated CpG Sites

Methylation changes in intronic and intergenic regions could significantly influence gene expression by affecting regulatory elements, while the presence of DMCpGs in promoters/TSSs and exons suggests potential direct impacts on gene transcription. In our study, the majority of DMCpGs were in intronic and intergenic regions, with fewer in promoters/TSSs, exons, and TTSs. This distribution suggests that methylation changes are more prevalent in non-coding regions, which could influence gene regulation indirectly [38,46].

The analysis of DMCpGs in testicular samples from mature male Senegalese sole revealed intricate relationships between methylation and gene expression. For instance, although the *sox* transcription factor gene family was identified through DMCpG annotation by proximity, this did not result in significant expression differences in the differential expression analysis. Previous findings in Oreochromis niloticus showed that *sox* genes have a stage-specific and sexually dimorphic expression, highlighting their complex roles in sex-linked functions. The overlapping distribution of *Sox* genes across tissues, their multifunctionality, and their functional overlap, along with species-specific genetic mechanisms and diverse sex-determining systems, contribute to the complexity and uncertainty in their functions [47].

On the other hand, the observed differences in methylation between mature F1 and wild groups, particularly in genes like *nanos1* and *egr1*, suggest that epigenetic mechanisms play a significant role in regulating gene expression. These mechanisms can both repress and enhance gene activity, highlighting their importance in the modulation of genetic functions. The higher methylation and down-regulation of these genes in the mature F1 group indicate a potential link between methylation and gene suppression. *Nanos1* showed up to six intergenic DMCpGs: one displaying a strong positive correlation (r = 0.9) and the others showing weaker negative correlations ranging from −0.2 to −0.6. Similarly, several DMCpG sites in both intronic and intergenic regions were associated with *err2a* and *esrrb*, showing positive (r = 0.5) and negative (r = −0.3) correlations, respectively.

Regarding the observed differences in methylation and expression between immature and mature wild groups, *GATA6* showed greater methylation and down-regulation in immature males, while *dmrt*2a exhibited lower methylation levels and was up-regulated in the same group. The varying correlation values between methylation and expression for *GATA6*, *dmrt*2b, and *dmrt*3b underscore the complexity and specificity of methylation’s impact. For example, *GATA6* had four neighboring DMCpGs with varying negative correlation values (r = −0.3 ~ −0.7), while intergenic DMCpGs associated with *dmrt*2b and *dmrt*3b exhibited strong negative correlations (r = −0.95 and r = −0.8). These findings suggest that methylation can influence gene expression in a gene-specific manner, and that its effects can differ significantly even among closely related genes.

In regard to the implication of the studied genes, *nanos1* has been identified in various fish species and plays a crucial role in germ cell differentiation [48]. A deficiency in this gene is associated with a reduction in primordial germ cells [49], suggesting its essential role in the migration of PGCs and preserving germline identity [50,51]. Additionally, previous studies have described the role of *GATA6* in gonadal development and reproduction in *C. semilaevis* [52] and the of roles of estrogen receptors (esr) in the molecular mechanism of sex determination and differentiation in *Hippoglossus hippoglossus* [53].

The *dmrt* transcription factor’s genes (double-sex and mab-3-related) are crucial for testes development and functioning [54,55]. In the flatfish *C. semilaevis*, *dmrt*1, *dmrt*2, and *dmrt*3 play significant roles in the maturation of male germ cells and the differentiation of gonads. Reference [56] found that the expression of these genes is inversely related to the methylation level of their promoters, indicating that methylation may regulate their expression and, consequently, their role in reproductive processes.

The methylation differences between two differently reared groups suggest that captive conditions may play a role in shaping methylation patterns. The significant correlation between methylation and expression indicates that these differences may affect certain biological processes and how they unfold in different-origin individuals. Our results also highlight complex dynamics regarding *dmrt* genes and their methylation/expression, the influence of other regulatory factors, as well as differences in how maturity affects the expression of these genes in wild-type and F1 individuals.

Similar differential methylation analysis performed in females resulted in different genes related to reproduction, to which different DMCpGs were associated by gene annotation. However, integration with transcriptomic data and correlation analysis did not show CpGs with a significant correlation (*p* < 0.05) neighboring these genes. These results highlight the distinct epigenetic mechanisms between males and females, potentially due to tissue specificity and the involvement of other regulatory factors [57]. This differentiation is crucial in understanding the reproductive dysfunctions observed in *Solea senegalensis*, which can impact the species’ reproductive success and sustainability.

### 4.3. Differential Gene Expression in Sex Differentiation and Reproductive Development

Significant differences in both methylation and gene expression were observed when comparing individuals of different sexes. This was evident in both the differentially expressed PCA and the MDS based on methylation differences measured in the fold change. Among samples of the same sex, distinct separation was observed between those with different rearing conditions. This separation was most notable in males when analyzing methylation differences, and in females when analyzing expression differences. The highest number of differentially expressed genes (DEGs) was observed between sexes, followed by differences in maturation status, and lastly, by differences between wild-type and F1 individuals. This pattern suggests that sex is the most influential factor in gene expression differences, while maturation and origin also play significant roles.

The differential gene expression analysis further highlighted substantial differences between males and females, with females generally showing higher expression compared to males. These differences were more pronounced in wild groups compared to F1 groups. Additionally, maturation significantly influenced gene expression within the same sex, with sex-specific changes. Wild individuals exhibited a larger shift in gene expression during maturation compared to F1 individuals. While sex seems like a primary factor when comparing gene expression and methylation patterns, with significant differences observed between males and females, maturation and origin (Wild vs. F1) also play important roles, especially in wild groups.

Particularly in genes related to reproduction and sex differentiation, the gene expression analysis revealed significant sex-specific differences; genes such as *gsdf*, *fshr*, and *amh* exhibited higher expression levels in male groups both immature and mature compared to female groups. This was further observed in *dmrt* and *sox* genes, indicating their crucial roles in male reproductive functions, such as testicular development and spermatogenesis. In contrast, *sox*19b, and hsd17b showed higher expression in females, indicating their involvement, such as in steroidogenesis, ovarian development, and general female reproductive processes [19,58].

The *gsdf* gene, which is vital for sex differentiation in fish, showed higher expression levels in immature males (both wild and F1) compared to mature groups, indicating its importance in the early stages of male development, and was nearly absent in females. This highlights its significant role in the sex differentiation processes of *S. senegalensis*. Additionally, the differential expression of the *fshr* and *amh* genes suggests their predominant role in the differentiation of male gonads. In teleosts, *gsdf* functions mainly in gonad differentiation, with its expression, regulation, and function varying significantly across species. It is usually expressed in Sertoli cells surrounding spermatogonia, suggesting its involvement in self-renewal, proliferation, and differentiation of spermatogonia [59]. This aligns with our findings, where *gsdf* showed higher expression in immature males, underscoring its role in early male development.

While the pathways and genes involved in sex differentiation are still not well-understood, and the reproductive challenges faced by hatchery-born individuals remain unresolved, the *fshr* gene was identified as playing a key role in sex determination in this species [19]. Although no significant differences in methylation or expression related to *fshr* were detected with the statistical criteria chosen for the group comparison analysis, genetic quantification reflected a lower expression in mature F1 males compared to wild-type males. Previously, Sambroni et al. (2013) studied FSH control of gene expression in fish both independently of and through steroid mediation [60]. This indicated that FSH can regulate gene expression via multiple pathways, which could explain the observed differences in *fshr* expression between wild and mature F1 males in *S. senegalensis*.

### 4.4. Methylation and Expression Analysis Integration

By integrating methylation coverage and expression analysis, we sought to identify significant correlations between CpG methylation and the expression of chosen key reproductive genes. Upon integrating methylation and expression data, significant positive correlations in CpGs neighboring *dmrt*1 (r = 0.99), *gnrh* (r = 0.95), and *cyp1b1* (r = 0.74) were observed in mature females. Only transcription factor *GATA6* showed a significant negative correlation (r = −0.68). These correlations were observed in intergenic and intronic regions, respectively.

In *C. semilaevis*, Liu et al. (2016) described the role of *GATA6* in gonadal development and reproduction [52]. While no notable differences were found in the total levels of methylation, the methylation patterns of certain sites varied among males, females, and pseudo males, with the latter showing a greater expression of *GATA6* in comparison to females. This study underscores the significant role of methylation in gene expression regulation. Similarly, our results highlight the crucial impact of methylation on gene expression, suggesting that epigenetic mechanisms are essential in regulating reproductive traits in Senegalese sole.

The absence of these genes when previously analyzing significant methylation differences between mature females suggests that while these genes are regulated by methylation, the differences in methylation levels between individuals may not be large enough to be detected as significant in a differential methylation analysis.

In contrast, the lack of significant correlations (r > 0.5) in male testes for selected genes indicates the influence of other regulatory factors in certain reproductive mechanisms, such as hormonal interactions [61]. These findings underscore the complex regulatory mechanisms governing reproduction in this species.

Our study further focused on genes with significant negative methylation/expression correlations to understand the sexual dysfunction observed in male sole. The enrichment analysis revealed significant enrichment of the MAPK, TGF-beta, and calcium signaling pathways. These pathways are crucial for cell differentiation, gonadal development, and signal transduction in maturation and cell activation processes, highlighting their importance in reproductive processes.

Gonadotropin-releasing hormone (GnRH) activates the MAPK cascade through the protein kinase A (PKA) signaling pathway, affecting gene expression, especially for GPα and LHβ, but not for FSHβ. This underscores the critical role of GnRH signaling in regulating reproductive hormones and processes [39,62,63,64]. In the DMCpG analysis of mature different-origin males, the MAPK pathway was associated with up-methylated CpGs in F1 males. Differential expression analysis showed a higher expression of *gnrh* in F1 groups, while *fshr* was more greatly expressed in mature wild males. De la Herrán et al. (2023) observed a consistent overexpression of the *fshr* gene in males at all developmental stages, from undifferentiated primordium to mature adults, indicating its crucial role in male reproductive development [19]. These results highlight the differences in reproductive gene regulation between wild and F1 males, with potential implications for their reproductive capabilities and success. This suggests differences in hormonal regulation between these groups, which could be due to environmental factors and the implication of potential epigenetic modifications. Furthermore, the GnRH signaling pathway was enriched when analyzing up-methylated genes in immature wild males versus the mature group, highlighting variations in methylation patterns within the MAPK pathway as fish transition from immaturity to maturity in individuals of different origins.

Moreover, when studying the methylation/expression correlation in mature male sole, receptors belonging to the OR1G1 family showed a significant positive correlation (r > 0.7). When analyzing this correlation between F1 and wild-type males independently, no significant differences were found. Chauvigné et al. (2016) found that mRNA for olfactory receptors and other reproduction-related transcripts, including cytochrome P450, show different expression levels in the upper olfactory epithelia of wild-caught and F1 sole [16]. This suggests that urine and intestinal fluid may play crucial roles in chemical communication during reproduction in sole, with the regulation at the olfactory epithelium level being sensitive to both the sex and maturity of the receiver. The physiological function of mature females’ urine in triggering the release of LH from the pituitary gland and enhancing spermatogenesis and milt production in males as previously described suggests that methylation may play a role in optimizing reproductive success.

Functional enrichment analyses further support the involvement of methylation in key biological processes and pathways linked to reproduction. These insights contribute to our understanding of the complex interplay between epigenetic modifications and gene expression in this species. Future studies could focus on tracking methylation and gene expression changes over time in the same individuals, providing insights into the dynamics of these processes. To address reproductive issues in cultured fish, it is crucial to integrate gene expression and methylation data with histological analysis, along with plasma hormone levels, vitellogenin, and other relevant assessments. Additionally, comparative studies across different environmental conditions, such as temperature changes, which have been shown to have an epigenetic influence, could elucidate how these factors affect methylation.

In the absence of further validation, we strictly considered the genes with significant differences and variations in methylation alongside a significant correlation. However, previous studies have shown that minor alterations in DNA methylation levels can act as drivers for the epigenetic regulation of gene expression. For instance, in Atlantic salmon, small changes in DNA methylation at CpG sites were found to correlate with transcriptional changes, suggesting that even minimal methylation variations can serve as regulatory epimarkers. Overall, our findings highlight the intricate nature of epigenetic regulation in reproductive mechanisms and emphasize the need for further research to fully understand the interplay between methylation and other regulatory factors in gene expression [38].

## 5. Conclusions

This study explores the methylome and gonadal transcriptome of *S. senegalensis*, focusing on the interactions of specific genes related to reproduction and the effects of sex, maturity, and rearing conditions. Our findings suggest that DNA methylation significantly regulates gene expression in *S. senegalensis*, acting as a mechanism that can both repress and enhance gene expression depending on the genomic context. The complexity of this epigenetic mechanism is evident from the varying levels of methylation and correlation rates, both positive and negative, observed in different DMCpGs associated with specific genes linked to reproduction. This variability could explain the differences in methylation and expression when comparing groups, as many genes show different associated DMCpGs with varying correlation rates.

Differential methylation comparisons revealed the highest number of DMCpG sites between the two maturation stages studied, followed by rearing conditions, and then between sexes. Additionally, the analysis of differential gene expression showed substantial differences between males and females, with females generally exhibiting higher expression levels. These differences were more pronounced in wild groups compared to F1 groups. The comparison between maturation stages revealed significant gene expression differences within the same sex, with wild individuals showing a larger shift during maturation compared to F1 individuals. While sex is a major factor in gene expression and methylation patterns, maturation and origin (Wild vs. F1) also play crucial roles, particularly in wild groups.

These findings suggest that methylation plays a crucial role in regulating gene expression in processes such as sexual differentiation and maturation. However, the influence of other factors on this regulation must also be considered. Our findings indicate that while rearing conditions affect methylation patterns and gene expression, these effects must be evaluated alongside other factors, such as sex-specific mechanisms during maturation. This underscores the complex interaction between epigenetic modifications and gene expression in Senegalese sole.

## Figures and Tables

**Figure A4:**
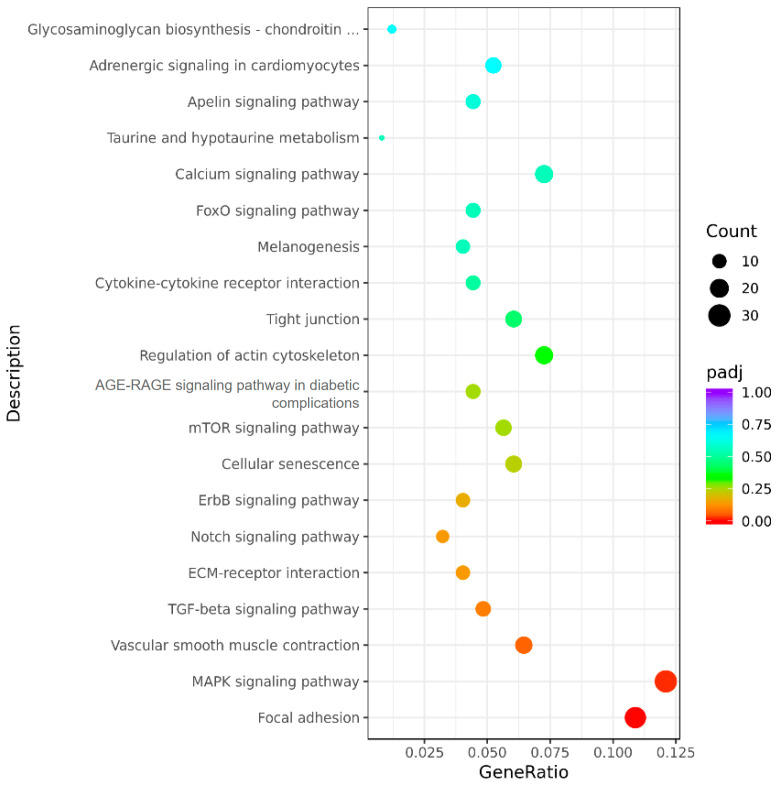
KEGG enrichment analysis scatter plot for genes neighboring DMCpGs that exhibited a significant negative correlation between methylation and gene expression in male gonadal tissue in *S. senegalensis*. Notes: The enrichment analysis scatter plot was generated using Novogene’s Novomagic platform. The Padj value, displayed on a color scale from 0 to 1, indicates the significance of enrichment. The gene ratio reflects the number of genes counted in relation to the total number of genes analyzed.

## Data Availability

The original contributions presented in this study are included in the article/Supplementary Material. Further inquiries can be directed to the corresponding author.

## Supplementary Materials

The following supporting information can be downloaded at https://www.mdpi.com/article/10.3390/biom15010054/s1, Table S1: Correlation rates between methylation coverage and gene expression in *Solea senegalensis*.

## Abbreviations

CpGs (CpG Sites); DMCpGs (Differently Methylated CpG Sites); cpm (Counts Per Million); DNMTs (DNA Methyltransferases); F1 (Filial 1); GnRH (Gonadotropin-Releasing Hormone); GO (Gene Ontology); IFF (Immature F1 Female); IFM (Immature F1 Male); IWF (Immature Wild Female); IWM (Immature Wild Male); KEGG (Kyoto Encyclopedia of Genes and Genomes); LogFC (Log2 Fold Change); MAPK (Mitogen-Activated Protein Kinase); MDS (Multidimensional Scaling); MFF (Mature F1 Female); MFM (Mature F1 Male); MWF (Mature Wild Female); MWM (Mature Wild Male); PCA (Principal Component Analysis); PCR (Polymerase Chain Reaction); qPCR (Quantitative Polymerase Chain Reaction); RRBS (Reduced Representation Bisulfite Sequencing); SD (Standard Deviation); TSS (Transcription Start Site); TTS (Transcription Termination Site).

## Appendix A. Tables

**Table A1:** Summary statistics for RRBS libraries and transcriptome sequencing data.

Sample	RRBS				RNA			
	Raw Bases (Gb)	Q20 (%)	Q30 (%)	GC (%)	Raw Bases (Gb)	Q20 (%)	Q30 (%)	GC (%)
Immature F1 Female	15.20	93.89	86.50	30.45	9.33	97.70	93.65	48.58
	11.70	93.34	85.79	32.96	6.11	97.71	93.52	49.37
	15.40	92.26	84.50	33.42	7.02	97.94	93.61	49.73
Immature F1 Male	18.70	93.90	86.29	30.61	7.08	97.40	92.86	47.62
	11.70	93.05	85.79	32.42	7.17	97.36	92.73	47.20
Immature Wild Female	15.30	93.49	86.17	33.69	7.00	97.80	93.30	49.22
	7.80	94.21	86.93	29.57	8.02	97.71	93.50	49.28
	13.10	92.26	84.47	34.07	8.85	97.78	93.37	48.94
Immature Wild Male	7.10	90.20	82.24	37.72	6.52	97.50	93.07	47.45
	5.00	92.79	85.32	32.44	9.90	97.35	92.75	47.55
	16.80	93.46	85.75	30.84	6.36	96.67	91.05	46.88
	29.50	93.76	86.40	31.78	7.59	97.50	93.06	47.40
Mature F1 Female	20.40	94.21	86.91	30.97	6.96	97.58	93.14	49.29
	16.90	93.76	86.15	30.43	7.46	97.77	93.48	48.98
	11.90	92.37	84.77	36.16	7.92	97.72	93.11	46.11
	13.00	92.45	84.78	31.68	8.17	97.43	92.62	48.36
Mature F1 Male	16.20	93.61	86.20	31.66	7.49	97.76	93.19	47.46
	13.20	93.09	85.57	31.66	7.41	97.32	92.63	47.10
Mature Wild Female	14.90	93.73	86.22	31.19	6.84	97.37	92.86	49.46
	18.60	93.56	85.72	29.53	17.60	97.83	93.71	48.35
Mature Wild Male	5.20	93.60	86.17	32.15	6.32	97.34	92.77	48.23
	8.40	92.86	85.08	31.15	6.67	97.56	93.21	47.56
	16.30	93.38	85.79	31.80	8.10	97.00	91.72	47.62
	4.60	89.72	81.48	37.68	6.42	97.42	92.94	47.75

Notes: “F1” stands for specimens produced by aquaculture. Raw Bases (Gb) indicates the mean raw bases in gigabases per sample within groups. Q20 (%) is the proportion of sequencing reads that meet a specified quality threshold of Q20 or higher per sample within groups. Q30 (%) is the proportion of sequencing reads that meet a specified quality threshold of Q30 or higher per sample within groups. GC Content (%) is the percentage guanine–cytosine content in the sequences per sample within groups.

**Table A2:** Summary of methylation calling results.

Sample	Cytosines Analyzed	Total Methylated Cs			Total C to T Conversions			C Methylated		
		CpG	CHG	CHH	CpG	CHG	CHH	CpG Context	CHG Context	CHH Context
Immature F1 Female	1,161,003,939	122,400,264	2,745,274	5,657,930	65,430,081	276,763,006	688,007,384	65.20%	1.00%	0.80%
	965,408,146	101,336,168	2,344,612	4,820,567	55,187,119	231,465,675	570,254,005	64.70%	1.00%	0.80%
	1,150,505,809	117,379,836	2,785,937	5,749,657	70,661,991	276,723,896	677,204,492	62.40%	1.00%	0.80%
Immature F1 Male	1,694,290,258	199,234,529	4,322,051	8,804,903	86,417,556	408,950,685	986,560,534	69.70%	1.00%	0.90%
	959,664,596	115,841,877	2,463,936	4,840,611	48,808,618	232,999,065	554,710,489	70.40%	1.00%	0.90%
Immature Wild Female	1,226,494,406	128,743,569	2,969,643	6,089,415	71,282,746	294,872,229	722,536,804	64.40%	1.00%	0.80%
	721,015,030	68,963,539	1,738,774	3,656,858	50,781,417	172,306,061	423,568,381	57.60%	1.00%	0.90%
	1,045,075,951	110,853,128	2,576,630	5,263,851	58,901,311	251,483,299	615,997,732	65.30%	1.00%	0.80%
Immature Wild Male	445,269,679	13,080,715	762,336	1,612,624	79,403,724	105,913,810	244,496,470	14.10%	0.70%	0.70%
	393,961,163	15,535,314	825,105	1,711,055	66,333,121	94,728,423	214,828,145	19.00%	0.90%	0.80%
	1,392,968,794	147,658,063	2,981,603	6,112,677	85,368,569	334,984,271	815,863,611	63.40%	0.90%	0.70%
	2,494,517,598	275,885,506	6,538,261	13,028,122	159,719,419	600,354,809	1,438,991,481	63.30%	1.10%	0.90%
Mature F1 Female	1,744,257,380	195,337,172	4,477,104	9,050,681	94,610,073	421,568,792	1,019,213,558	67.40%	1.10%	0.90%
	1,379,217,695	144,087,159	3,236,233	6,724,657	81,855,177	330,057,506	813,256,963	63.80%	1.00%	0.80%
	883,508,418	96,889,871	2,147,202	4,356,367	48,382,792	213,161,844	518,570,342	66.70%	1.00%	0.80%
	870,898,914	87,334,144	1,817,947	3,782,783	54,461,551	207,601,653	515,900,836	61.60%	0.90%	0.70%
Mature F1 Male	1,268,863,036	103,606,717	2,818,802	5,648,044	127,480,409	304,775,418	724,533,646	44.80%	0.90%	0.80%
	937,562,250	99,915,441	2,223,903	4,271,133	64,136,781	226,344,652	540,670,340	60.90%	1.00%	0.80%
Mature Wild Female	1,295,247,927	131,381,708	3,150,768	6,664,051	74,313,745	308,977,506	770,760,149	63.90%	1.00%	0.90%
	1,570,474,527	157,303,960	3,295,521	7,050,272	93,007,036	372,067,275	937,750,463	62.80%	0.90%	0.70%
Mature Wild Male	427,928,286	18,155,112	875,090	1,814,840	69,550,316	105,323,455	232,209,473	20.70%	0.80%	0.80%
	684,891,178	24,904,912	1,321,040	2,757,633	113,021,414	166,103,962	376,782,217	18.10%	0.80%	0.70%
	1,340,369,536	139,498,190	2,850,138	5,715,888	86,932,484	322,592,496	782,780,340	61.60%	0.90%	0.70%
	272,155,711	8,534,487	468,304	979,457	47,311,224	65,128,833	149,733,406	15.30%	0.70%	0.60%

Notes: Summary of methylation calling results in *S. senegalensis* for eight study groups. “F1” stands for specimens produced by aquaculture. The reported cytosine counts are presented for the CpG, CHG, and CHH contexts (where H represents C, T, or A).

**Table A3:** Performed comparisons between groups in *Solea senegalensis* for differential methylation analysis.

GROUP	IFM	IWF	IWM	MFF	MFM	MWF	MWM
IFF	IFF/IFM	IFF/IWF		IFF/MFF			
IFM			IFM/IWM		IFM/MFM		
IWF			IWF/IWM			IWM/MWF	
IWM							IWM/MWM
MFF					MFF/MFM	MFF/MWF	
MFM							MFM/MWM
MWF							MWF/MWM

Notes: Group comparisons keys are shown as follows: MFM/MWM (Mature F1 Male vs. Mature Wild Male), IFM/IWM (Immature F1 Male vs. Immature Wild Male), IWM/MWM (Immature Wild Male vs. Mature Wild Male), IFM/MFM (Immature F1 Male vs. Mature F1 Male), MFF/MWF (Mature F1 Female vs. Mature Wild Female), IFF/IWF (Immature F1 Female vs. Immature Wild Female), IWF/MWF (Immature Wild Female vs. Mature Wild Female), IFF/MFF (Immature F1 Female vs. Mature F1 Female), MFF/MFM (Mature F1 Female vs. Mature F1 Male), IFF/IFM (Immature F1 Female vs. Immature F1 Male), MWF/MWM (Mature Wild Female vs. Mature Wild Male), and IWF/IWM (Immature Wild Female vs. Immature Wild Male). To maintain consistency and order in the comparisons, the following groups were established: F1 vs. Wild, Immature vs. Mature, and Females vs. Males. While the order of the groups does not affect the results, it is important to consider this order when interpreting the findings.

**Table A4:** Gene expression levels measured as gene counts for reproduction-related genes across the studied groups in *Solea senegalensis*.

Gene	MWM	IWM	MFM	IFM	MWF	IWF	MFF	IFF	SD
*gsdf*	20,585.25	22,900.00	28,574.00	32,425.00	57.50	56.33	49.50	50.00	14,371.75
*sox19b*	28.50	43.75	65.50	105.00	9985.50	7506.67	6473.50	7789.00	4320.61
*fshr*	9677.00	6814.75	6331.50	6229.00	7.50	9.67	5.25	11.00	4021.85
*dmrt1*	1537.25	1178.25	1220.50	1422.50	14.00	5.67	6.50	8.67	720.01
*sox9*	1513.75	1072.75	1294.00	1076.50	13.00	38.67	51.50	40.33	657.87
*hsd17b*	147.00	151.25	111.50	116.00	1338.50	1198.00	1197.25	1438.00	625.73
*GATA6*	1458.50	737.50	992.50	927.00	19.50	8.33	112.00	17.67	566.51
*dmrt3a*	648.25	461.00	853.00	472.50	208.50	131.00	146.50	133.67	271.87
*gnrh*	113.75	221.50	363.50	307.50	2.50	1.00	4.50	2.00	151.01
*amh*	233.75	252.25	310.00	401.00	148.50	41.67	48.00	56.00	134.40
*cyp1b1*	287.50	172.25	168.50	222.50	3.00	2.33	65.00	2.67	112.00
*nanos1*	351.25	167.00	220.50	211.50	73.50	37.00	151.50	31.00	108.29
*dmrt2*	113.75	137.75	348.00	229.00	178.50	41.00	62.50	37.67	106.60
*sox3*	168.75	260.25	198.00	175.00	42.50	32.67	22.75	25.67	94.87
*sox8*	250.00	32.50	35.00	46.50	32.50	10.33	40.50	12.33	78.82
*sox6*	30.50	23.25	10.00	18.50	4.50	3.67	6.00	1.67	10.58

Notes: Group keys are shown as follows: MWM (Mature Wild Male), IWM (Immature Wild Male), MFF (Mature F1 Female), IWF (Immature Wild Female), IFF (Immature F1 Female), IFM (Immature F1 Male), MFM (Mature F1 Male), and MWF (Mature Wild Female); Standard Deviation (SD).
